# Tubing-Free Microfluidic Microtissue Culture System Featuring Gradual, *in vivo*-Like Substance Exposure Profiles

**DOI:** 10.3389/fbioe.2019.00072

**Published:** 2019-04-02

**Authors:** Christian Lohasz, Olivier Frey, Flavio Bonanini, Kasper Renggli, Andreas Hierlemann

**Affiliations:** ^1^Bioengineering Laboratory, Department of Biosystems Science and Engineering, ETH Zürich, Zurich, Switzerland; ^2^InSphero AG, Schlieren, Switzerland

**Keywords:** microfluidics, tilting chip, microtissues, pharmacokinetics, drug dosing

## Abstract

*In vitro* screening methods for compound efficacy and toxicity to date mostly include cell or tissue exposure to preset constant compound concentrations over a defined testing period. Such concentration profiles, however, do not represent realistic *in vivo* situations after substance uptake. Absorption, distribution, metabolism and excretion of administered substances in an organism or human body entail gradually changing pharmacokinetic concentration profiles. As concentration profile dynamics can influence drug effects on the target tissues, it is important to be able to reproduce realistic concentration profiles in *in vitro* systems. We present a novel design that can be integrated in tubing-free, microfluidic culture chips. These chips are actuated by tilting so that gravity-driven flow and perfusion of culture chambers can be established between reservoirs at both ends of a microfluidic channel. The design enables the realization of *in vivo*-like substance exposure scenarios. Compound gradients are generated through an asymmetric Y-junction of channels with different hydrodynamic resistances. Six microtissues (MTs) can be cultured and exposed in compartments along the channel. Changes of the chip design or operation parameters enable to alter the dosing profile over a large range. Modulation of, e.g., the tilting angle, changes the slope of the dosing curves, so that concentration curves can be attained that resemble the pharmacokinetic characteristics of common substances in a human body. Human colorectal cancer (HCT 116) MTs were exposed to both, gradually decreasing and constant concentrations of Staurosporine. Measurements of apoptosis induction and viability after 5 h and 24 h showed different short- and long-term responses of the MTs to dynamic and linear dosing regimes

## Introduction

In the last decades, microphysiological systems (MPSs) have been proven to better mimic human *in vivo* physiology in *in vitro* cell cultures. MPSs, often referred to as “organs on chips,” are *in vitro* platforms designed to model the spatial, chemical, structural, and physiological elements of *in vivo* cellular environments. In most cases, they include a combination of advanced cell culture models and microfluidic technology. In the last couple of years, a plethora of new, specialized systems have been presented by both, academia and industry (Marx et al., 2016). The list of different systems ranges from single- to multi-organ platforms and include tissues in healthy or diseased states (Zhang et al., 2018). All these systems commonly include defined microchannel structures that enable fluidic interconnection. Perfusion enables to (i) precisely control the microenvironment directly at the cell culture site, (ii) constantly deliver nutrients, oxygen, and other substances, (iii) establish a communication route between individual modules, i.e., organs or microtissues, and (iv) wash away cellular waste products. A precise liquid-flow and temperature control, as well as the possibility to very closely mimic *in vivo* situations make these tools very promising candidates for efficacy and/or toxicity testing of substances for the pharmaceutical industry, in particular as they allow to investigate processes in a more systemic way (Marx et al., 2016; Wang et al., 2018). As those systems become better and better in reproducing *in vivo* situations in the human body, one of the remaining challenges that has been largely neglected in setting up an efficacy and toxicity screening pipeline is pharmacokinetics (PK).

**Figure 1:**
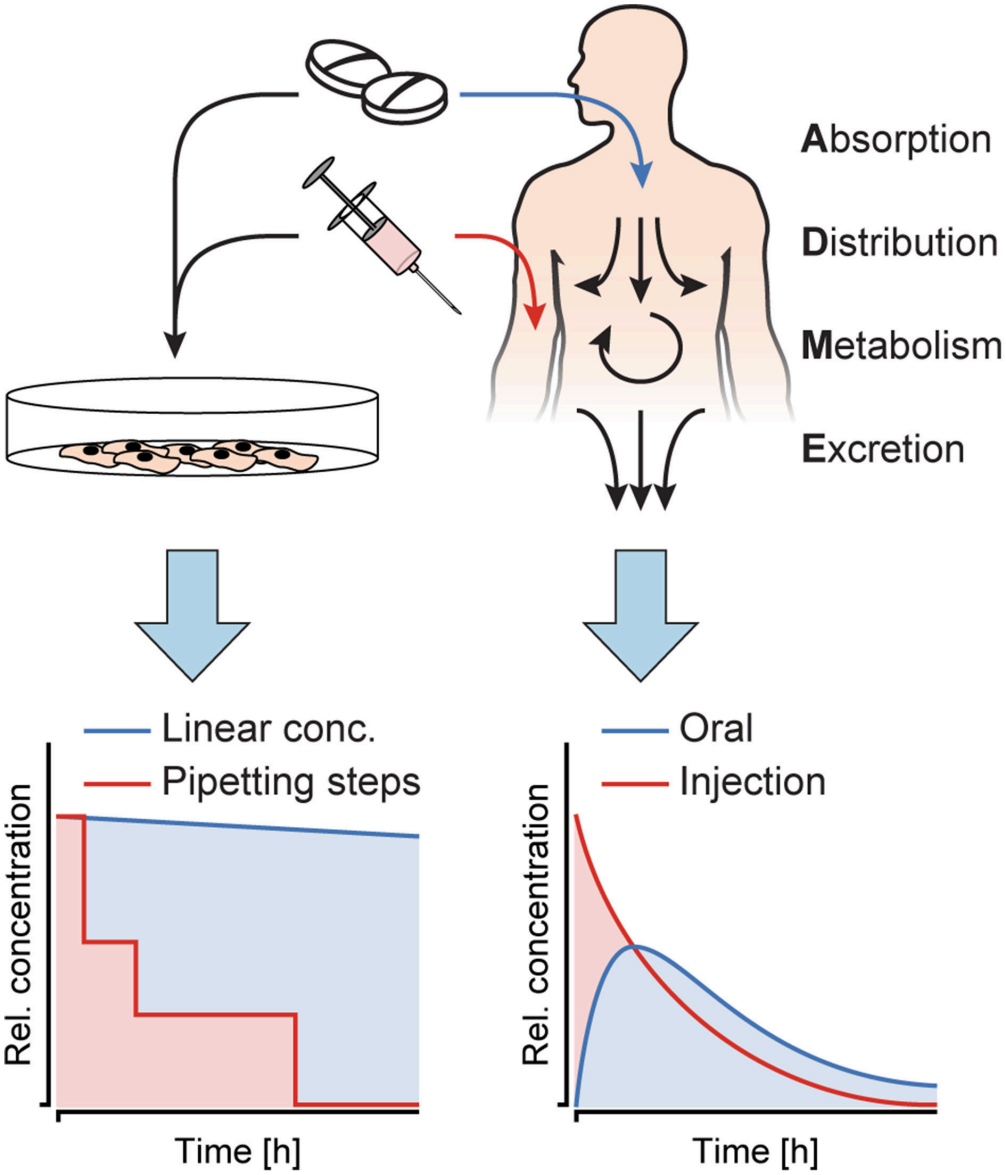
Substance concentration profiles in *in vitro* experiments as opposed to *in vivo*. Cell culture models *in vitro* are exposed to constant concentrations or sudden substance concentration steps generated by pipetting, while in the human body, the interplay of absorption, distribution, metabolism and excretion (ADME) produces distinct, dynamic substance concentration profiles upon oral administration or injection of a substance.

PK describe the fate of a substance, once it enters the human body. Upon entering, there are four key processes that happen with the substance (Figure 1): (i) absorption into the body through an epithelial barrier, (ii) distribution of the substance within the body or certain tissues, (iii) metabolization of the substance, and (iv) excretion of the substance itself and/or its metabolites. These four processes are summarized by the acronym ADME, and their interplay results in distinct concentration profiles over time in the human body, which highly depend on the nature of the substance and its administration. The concentration dynamics of a substance can also impact its efficacy and/or toxicity, since those dynamics define the exposure duration above a specific effective/toxic threshold concentration (Saif et al., 2009; Sejoong et al., 2016). While *in vitro* model systems have been used to extract specific ADME parameters, only very few systems allow for exposure of target organ models to such dynamic concentration profiles of a substance of interest. On the contrary, *in vitro* studies routinely include dosage of constant substance concentrations over a defined time, which are not representative of the *in vivo* situation. Figure 1 illustrates the differences between (left) *in vitro* substance concentration profiles, which are mostly linear, or, in some cases, more complex and then realized through precisely-timed, stepwise pipetting, and (right) dynamically and gradually changing *in vivo* concentration profiles. Studies showed that simplified protocols often lead to false positive results during toxicity and efficacy testing (Tsaioun et al., 2016).

To attain an improved representation of pharmacokinetics in *in vitro* systems, there are two major thrusts that are currently pursued and implemented. In a first thrust, a majority of the relevant organs that are involved in ADME are included and inter-linked in a single chip or microfluidic system (Sung et al., 2010; Maass et al., 2017; Edington et al., 2018). Substantial efforts are required for finding the appropriate organ ratio and for combining all organ surrogates so that they are functional at the same time in a common culture medium. This approach, therefore, entails very high biological complexity (Prantil-Baun et al., 2018), which may compromise experimental reproducibility and throughput. In a second approach, pharmacokinetic concentration profiles are generated through rather complex external devices, for example, by a combination of multiple precision pumps that enable a defined temporal modulation of the substance concentration for specific single organs of interest (Wikswo et al., 2018). There have been several attempts to use such systems and apply spatial and temporal gradients of substances on biological samples, such as Xenopus embryonic tissue (Kim et al., 2011), or lymphoid tissue explants (Ross et al., 2017). Further, Song et al. (2018) investigated exposure of single cells to pulsatile and gradually ramped concentration profiles of growth factors. Another solution to expose cancer cells to pulses of tumor necrosis factor (TNF) without the need for external pumps, has been presented by Lee et al. (2016). Perfusion in their chip was gravity-driven, and pulses of TNF were induced by manually elevating the dosing reservoir.

Here, we developed a new approach to generate dynamic substance concentration profiles in microfluidic cell culture systems. The proposed design and working principle can be integrated into tubing-free microfluidic platforms, which feature gravity-driven flow perfusion upon platform tilting. The specific new design feature consists of an asymmetric Y-junction, connected to three medium reservoirs (Lohasz et al., 2017). Upon repeated tilting of the chip, the liquids from the reservoirs mix, which gradually changes the substance concentration at the cell culture sites. The slope of the concentration profile can be modulated by changing the channel dimensions, the initial volume of the substance solution, or the tilting angle.

As a proof on concept, we integrated the new design feature into a microfluidic microtissue culture chip with 6 compartments that was similar to a previously presented approach (Kim et al., 2015a; Lohasz et al., 2018). Human colorectal tumor microtissues (HCT 116) were loaded into the MT compartments of the chip and exposed to Staurosporine. Staurosporine features strong, promiscuous kinase inhibitor properties by competitive binding to the ATP-binding site of kinases (Karaman et al., 2008). Therefore, it is frequently used as an anti-cancer tool compound for *in vitro* studies. The tumor microtissues (TuMTs) were exposed to a physiologically relevant concentration profile, which resembled that after an injection of a substance in a human body. This profile could be recreated by the chip system. The results were compared to constant-concentration drug dosing schemes. We saw that the TuMTs responded differently to *in vivo*-like drug dosing with decreasing concentrations in comparison to traditionally applied, constant concentrations.

## Materials and Methods

### Design Principle

**Figure 2:**
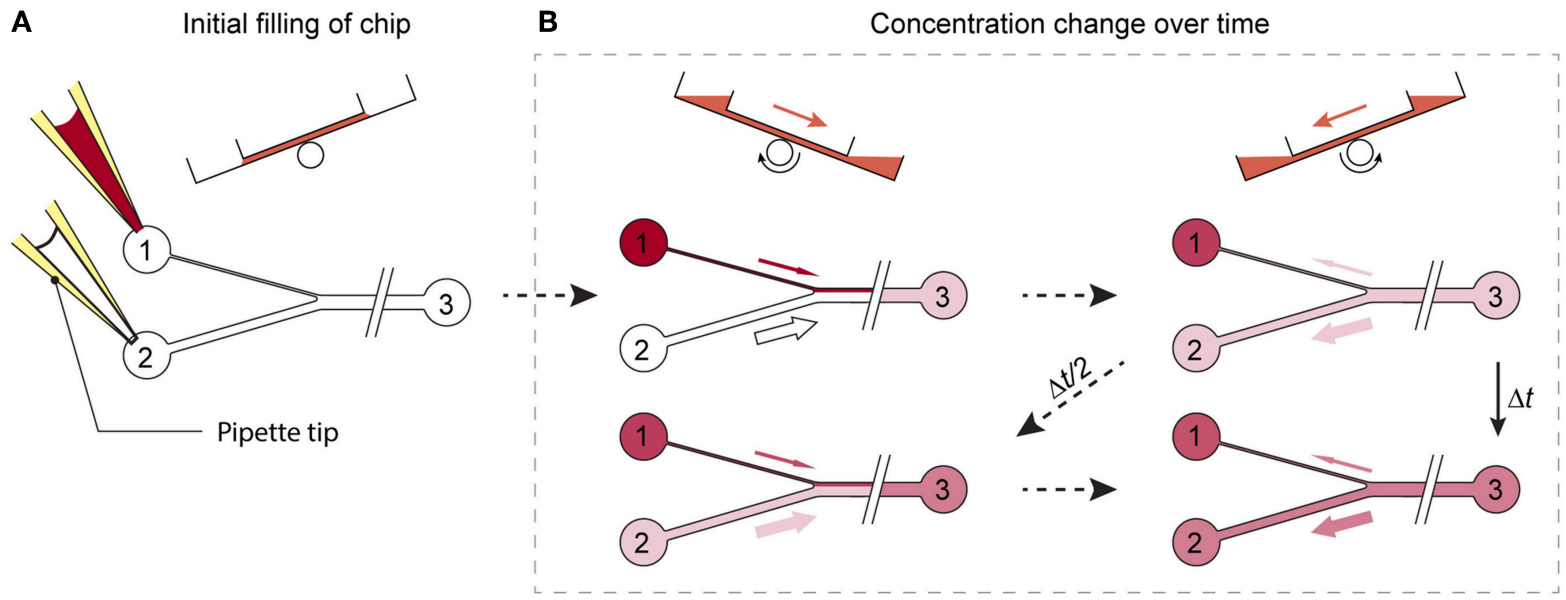
Working principle of the design. **(A)** Reservoirs 1 and 2 are filled with substance-loaded and plain cell culture medium. **(B)** Upon tilting of the chip, unequal volumes of liquid flow through the differently sized channels. While the substance concentration is, with each tilting interval Δ*t*, gradually enriched in reservoirs 2 and 3, the substance is diluted in reservoir 1, until a concentration equilibrium is reached.

A microfluidic channel design was developed, that enables a gradual change of substance concentrations over time at the cell or MT culturing site. Prerequisite for the integration of the proposed design feature is a chip system with medium reservoirs at both ends of a main channel and a flow actuation upon tilting of the system. Cell or MT models are cultured in compartments along the main channel and perfused upon tilting the chip back and forth. The specific new design feature consists of an asymmetric Y-junction, which is connected to three medium reservoirs, two reservoirs at one end of the main channel and a third reservoir at the other end of the channel (see Figure 2).

To produce a gradually changing concentration profile in the main channel, the two reservoirs left of the Y-junction in Figure 2A are filled with two different medium compositions, e.g., plain cell culture medium and medium containing a substance of interest. Upon tilting of the chip so that the 2 reservoirs left of the Y-junction are higher, the two liquids in the reservoirs start flowing and combine in the Y-junction to flow along the common main channel. The different cross-sections of the two left channels entail different hydrodynamic resistances and different flow rates through the two channels into the main channel. Upon tilting in the opposite direction so that the reservoir at the right side is higher, a flow backwards to the Y-junction is initiated. The liquid in the main channels then splits up at the Y-junction at the same volume ratio at which it entered the common channel before. Repeated tilting of the device with a defined tilting angle slowly mixes the media originating from the two reservoirs at the left side over time, until an equilibrium in the main channel is reached (Figure 2B). If, for example, a substance is added into the reservoir that is connected to the channel featuring the lower flow rate, and plain cell culture medium is applied to the other one, the substance concentration in the main channel is gradually enriched over time (Figure 2B).

**Figure 3:**
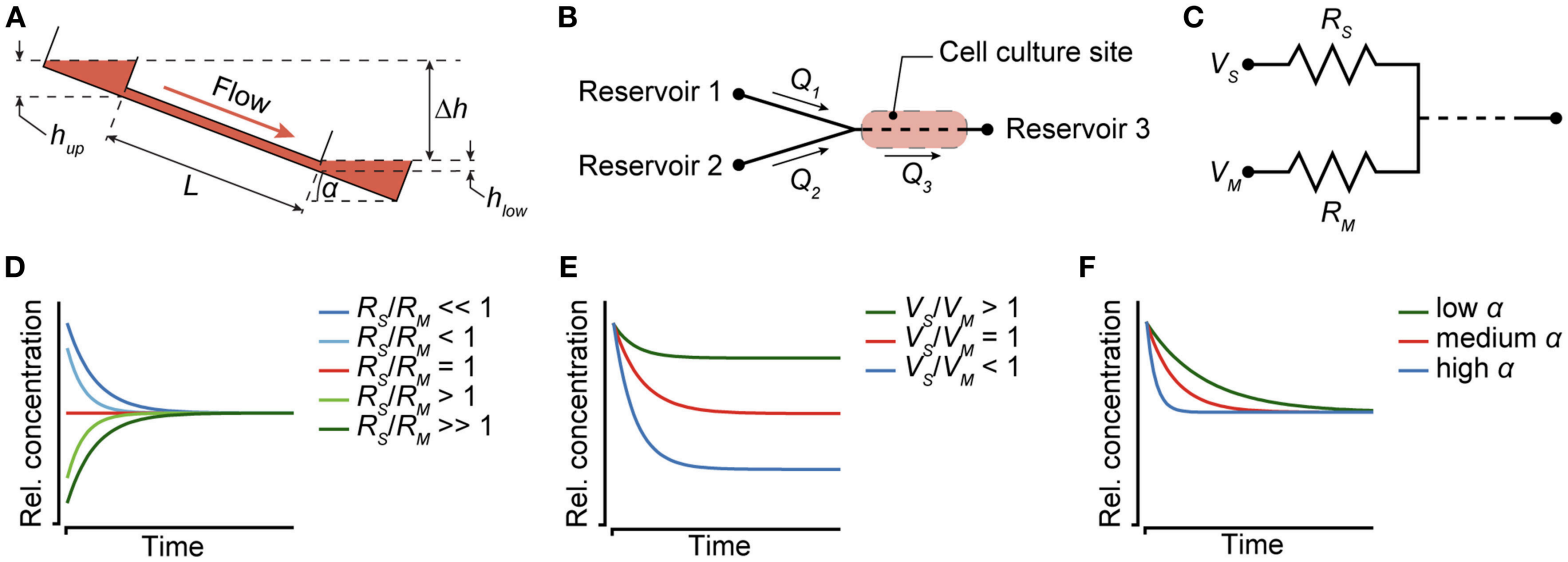
Modulation of concentration profiles. **(A)** Flow in the chip is dependent on the tilting angle α and the resulting height difference Δ*h* between the liquid levels in the reservoirs at the left and right side. **(B)** Two reservoirs that are connected to channels with unequal flow rates *Q*_*1*_ and *Q*_*2*_ feed into a Y-junction. The flow-rate difference can be generated by different channel widths or dimensions. A cell culture site is located between the Y-junction and a third reservoir, and can be subjected to dynamic substance concentration profiles. **(C)** The channel resistances *R*_*S*_ and *R*_*M*_, the initially applied liquid volumes *V*_*S*_ and *V*_*M*_, and the tilting angle α can be used to modulate the concentration profiles. **(D)** The ratio of resistances can be used to define the time, after which an equilibrium is reached (for constant *V* and α). **(E)** The ratio of input volumes in reservoirs 1 and 2 defines the concentration, at which an equilibrium is reached (for constant *R* and α). **(F)** The tilting angle α modulates the slope of the concentration profile (for constant *R* and *V*).

The change in the substance concentration in the main channel depends on the different flow rates through the channels at the left side and the resulting total liquid volumes that are fed into the main channel per tilting interval (Figure 3B). Flow rates, in turn depend on the height difference or hydrostatic pressure difference between the upper and lower reservoirs of the tilting system. The tilting angle α and the distance *L* between the opposite ends of the channel define the height difference Δ*h* between the two reservoirs, to which the height of the liquid column in the upper reservoir *h*_*up*_ is added and from which the liquid column height in the lower reservoir *h*_*low*_ is subtracted.

(1) $$\begin{array}{l} \Delta h\;=\;L\cdot sin\alpha \;+h_{up}-h_{low} \end{array}$$

In a rectangular channel, the hydrodynamic resistance *R*_*h*_ is dependent on the liquid viscosity and the channel dimensions, as described by Bruus (2008):

(2) $$\begin{array}{l} R_{h}\approx \frac{12\cdot \eta \cdot L}{{w\cdot h}^{3}\cdot \left(1-0.63\cdot \frac{h}{w}\right)} \end{array}$$

with η denoting the liquid viscosity and *L, w*, and *h* (*w* > *h*) describing the length, width and height of the channel, respectively.

Using the tilting-dependent hydrostatic pressure difference

(3) $$\begin{array}{l} \Delta P=\rho \cdot g\cdot \Delta h \end{array}$$

and the hydrodynamic resistance *R*_*h*_ (equation 2) of each section of the channel, the volumetric flow rates *Q* in each section of the channel can be calculated as:

(4) $$\begin{array}{l} Q=\frac{\Delta P}{R_{h}}\cdot \end{array}$$

The flow rates in the three channels connected to the Y-junction then are determined by:

(5) $$\begin{array}{l} Q_{1}+Q_{2}=Q_{3} \end{array}$$

with *Q*_1_ and *Q*_2_ referring to the differently sized channels joining into the common channel featuring a flow rate *Q*_3_ (Figure 3B).

To establish unequal flow rates in the two channels left of the Y-junction, their hydrodynamic resistances or geometric dimensions can be adjusted. Thus, a change of dimensions, such as the channel widths of the two channels, allows for simple adjustment of their specific hydrodynamic resistances. Figure 2 shows a schematic view of the microfluidic-channel layout with an integrated, asymmetric Y-junction. To predict the substance concentration in the common channel, the dilution equation:

(6) $$\begin{array}{l} C_{x}\cdot V_{x}=C_{y}\cdot V_{y} \end{array}$$

was used and adjusted to calculate the concentration resulting from mixing three liquids:

(7) $$\begin{array}{l} C_{3}(t)=\frac{C_{1}\cdot V_{1}(t)+C_{2}\cdot V_{2}(t)+C_{3}(t_{0})\cdot V_{3}(t_{0})}{V_{1}(t)+V_{2}(t)+V_{3}(t_{0})}. \end{array}$$

*C*_3_ refers to the concentration in the common channel and reservoir 3 at time point *t* after tilting of the chip. *C*_1_ and *C*_2_ represent the concentrations of the liquids in the differently sized channels upstream of the Y-junction, and *V*_1_ and *V*_2_ are the liquid volumes flowing into the main channel during time *t*. *C*_3_*(t*_0_*)* and *V*_3_*(t*_0_*)* denote the concentration and volume of the liquid phase already present in the main channel before initiation of the tilting cycle. The liquid volumes V_1_ and V_2_ can further be described by:

(8) $$\begin{array}{l} \begin{array}{l} V_{1}(t)=Q_{1}\cdot t,\;and\\ V_{2}(t)=Q_{2}\cdot t \end{array} \end{array}$$

with *t* denoting the time, during which the flow rates *Q*_1_ and *Q*_2_ are present in the two differently sized channels and during which the liquid flows into the common channel.

Equation (7), combined with equations (4) and (8), was then used to derive the time-dependent recurrence relation for calculating the concentrations in the system, when the chip is tilted toward reservoir 3:

(9) $$\begin{array}{l} C_{3}\left(t+\Delta t\right)=\frac{C_{1}\left(t\right)\cdot Q_{1}\cdot \Delta t+C_{2}\left(t\right)\cdot Q_{2}\cdot \Delta t+C_{3}\left(t0\right)\cdot V_{3}\left(t\right)}{Q_{1}\cdot \Delta t+Q_{2}\cdot \Delta t+V_{3}\left(t\right)} \end{array}$$

When tilting the chip into the opposite direction, the concentrations in reservoirs 1 and 2 can be calculated using:

(10) $$\begin{array}{lll} C_{1}\left(t+\Delta \frac{t}{2}\right) & = & \frac{C_{3}\left(t\right)\cdot Q_{3}\cdot \Delta t+C_{1}\left(t\right)\cdot Q_{1}\cdot \Delta t}{Q_{3}\cdot \Delta t+Q_{1}\Delta t}\;,\;and \end{array}$$

(11) $$\begin{array}{lll} C_{2}\left(t+\Delta \frac{t}{2}\right) & = & \frac{C_{3}\left(t\right)\cdot Q_{3}\cdot \Delta t+C_{2}\left(t\right)\cdot Q_{2}\cdot \Delta t}{Q_{3}\cdot \Delta t+Q_{2}\cdot \Delta t}. \end{array}$$

Here, Δ*t* denotes the interval between two successive tilting cycles into the same direction, and Δ*t/2* between two tilting steps into the opposite direction (Figure 2B). *C*_1_*(t), C*_2_*(t)*, and *C*_3_*(t)* are the time-dependent concentrations in reservoirs 1 to 3, and *V*_3_*(t*) is the time-dependent liquid volume within the main channel and in reservoir 3. At *t* = *0*, all channels are assumed to be filled with plain cell culture medium. Equations (9–11) allow for simple calculations of the analyte concentration in the main channel and in the reservoirs at discrete times.

### Chip Fabrication

The chip was fabricated using polydimethylsiloxane (PDMS) and conventional soft lithography methods. The bottom structures comprised the channel structures and were fabricated using a two-layer SU-8 master on a 4-inch silicon wafer (Supplementary Figure 1). For the first layer with the perfusion channel designs, SU-8 100 photoresist (Microchem Corp., Newtown, MA, USA) was spin-coated onto the wafer in a 100-μm thick layer and was exposed to UV through a transparency mask featuring the designed pattern. The second 25-μm-thick layer of SU-8 25 photoresist (Microchem Corp.) comprising the mixing structures was coated on top of the first layer and was exposed a second time to UV though a transparency mask.

For the top structures of the chip, a positive mold of the chip was designed and 3D-printed (Protolabs, Feldkirchen, Germany). The structures comprised the top part of the MT compartments and the reservoirs. The positive mold was then used to cast a flexible, negative mold out of PDMS (Dow Corning, Midland, MI, USA). A 7:1 ratio of polymer and curing agent were used for better stability, and the cast was cured at room temperature for 24 h to reduce heat-induced shrinking of PDMS as much as possible (Lee and Lee, 2008). The SU-8 bottom mold and the PDMS top mold were then placed in a desiccator and treated with trichloro(1H, 1H, 2H, 2H-perfluoroctyl)silane (Sigma-Aldrich Chemie GmbH, Buchs, Switzerland) to prevent fusion with the final chip and to enable multiple usage of both molds.

Designated alignment structures enabled the easy and fast alignment of the two molds, before PDMS was filled and cured in a vacuum oven at 80°C for 4 h. Subsequently, the casted PDMS devices were removed from the molds and cut into their final size of 23 mm × 73 mm. To close the channels at the bottom side, the PDMS devices were O_2_-plasma-bonded to microscopy glass slides that were previously spin-coated with a thin layer of PDMS (30 s, 2,000 rpm). The PDMS layer on the glass slide was needed to achieve uniform coating properties of all four channel surfaces. The glass slides enabled easy device handling and use with standard inverted microscopy setups. After the assembly, all channels and surfaces to be exposed to liquids or cells were coated with Biolipidure 206 (NOF America Corporation, White Plains, NY, USA) to render them hydrophilic and to prevent MTs from attaching to the surfaces.

### Device Operation

The devices were operated in a handling frame (Microfluidic ChipShop, Jena, Germany) holding four chips in parallel. To limit evaporation, a custom-cut adhesive polyester film with small holes for oxygen exchange was attached to cover the MT compartments and the medium reservoirs. Further, a lid (Microfluidic ChipShop), fitting to the handling frame, was used to close the chips and enabled stacking of multiple frames.

Channels and reservoirs were initially flooded with plain cell culture medium to a level that standing drops formed within the top rim of the MT compartments. Throughout the experiment, these standing drops remained stable at the top rim structure of the MT compartments. Surface tension stabilized the standing drops, which resulted in a constant volume in the compartment. Next, preformed spherical MTs were transferred into the corresponding compartments. They were harvested from a 96-well microtiter plate with a pipette. After sedimentation of the MTs to the opening of the pipette tip, the tip was brought into contact with the standing drop. The MT then sedimented down—driven by gravity—to the bottom of the designated compartment.

Before starting an experiment, the frame was tilted along its long axis with the branched side facing down while the upper reservoir on the other side was completely emptied of medium. The channels did not completely drain due to capillary forces at the connections to the reservoirs. The medium in the two lower reservoirs was then removed and replaced with the required volume of plain medium and medium containing a substance of interest at a predefined concentration. The reservoirs could hold a maximum volume of 100 μL. To achieve dosing curves with increasing concentration, the substance was administered into the reservoir connected to the narrow channel. For dosing curves featuring decreasing concentrations, the reservoir connected to the wide channel was loaded with the substance. After loading, repetitive tilting was started and the experiment began.

The assembled and loaded handling frame was operated on a programmable tilting stage (InSphero AG, Schlieren, Switzerland), as shown in Supplementary Figure 2. The stage allows for precise adjustment of positive and negative tilting angles, for defining resting times at tilted or horizontal positions and transition times between positive and negative angles. The entire experimental setup including the tilting stage and a stack of handling frames was operated in a standard cell-culture incubator at mammalian cell culture conditions (37°C, 5% CO_2_, 95% humidity).

### Measurements of Concentration Curves

To track concentration curves over time, cell culture medium containing amaranth red dye (Sigma-Aldrich, Buchs, Switzerland) was used to mimic a drug solution. 100 μL of the dye solution were loaded into one reservoir at the branched side of the channel and 100 μL of plain cell culture medium were added to the other one at the same side of the chip. While repeatedly tilting the chip, 1 μL of the mixed liquid was removed from the shared reservoir on the other side of the chip, and its absorbance at a wavelength of 520 nm was measured in reference to the initially loaded solution of amaranth red using a NanoDrop2000 (Life Technologies Europe B.V., Zug, Switzerland).

For the realization of concentration profiles that resembled *in vivo* concentrations after injection of a substance, cell culture medium containing 15 μM 5-fluorouracil (5-FU; Sigma-Aldrich) was used, along with plain cell culture medium in the chip. To measure the concentration profile, medium-filled chips with and without tumor microtissues (TuMTs) were used. 25 μL of drug-containing medium were loaded into the reservoir, connected to the wide channel, and 75 μL of plain medium were loaded into the reservoir with the narrow channel. The concentration profile was induced by tilting the chip at a tilting angle of 20° with waiting times of 2 min and 30 s at the tilted positions and transition times of 30 s. Samples of 10 μL were taken from the common reservoir after 3, 15, 33, 63, 123 min and after 24 h. For each time point, individual chips were used. The samples were diluted with 40 μL of plain medium and snap-frozen on dry ice. 5-FU was externally quantified by ultra-performance liquid chromatography, coupled to mass spectrometry UPLC-MS (Admescope Oy, Oulu, Finland).

### Cell Culturing

HCT 116 colorectal tumor cells (ATCC®, LGC Standards GmbH, Wesel, Germany) were used for cell culture experiments. The cells were maintained in RPMI-1640 basal medium (Gibco, Fisher Scientific, Illkirch Cedex, France), supplemented with 10% fetal bovine serum (FBS, Sigma-Aldrich) and 1% penicillin/streptomycin (Life Technologies Europe B.V.). Cells were passaged at 80% confluency. To form MTs with a diameter of 200–250 μm, 70 μL of medium, containing 300 cells, were transferred into each well of an Akura™ 96-well plate (InSphero AG) and centrifuged for 2 min at 250 × g. The plate was then placed in a tilted position in an incubator to collect cells in one corner of the wells and to promote MT formation. Cells were used for further experiments after 96 h.

### Dosing Experiments

For physiological dosing experiments, previously formed and equally sized MTs were transferred into the six MT compartments of the microfluidic chip. Briefly, MTs were aspired together with 3 μL of cell culture medium using a standard micropipette. Upon sedimentation of the MT by gravity to the opening of the pipette tip, the MT was transferred into the MT compartment by contact transfer.

To produce gradually decreasing concentrations over time, which are representing concentration profiles, e.g., upon intravenous injection of a drug, 25 μL of the drug solution containing the maximum concentration (C_max_ = 0.75 μM Staurosporine) were loaded into the reservoir, connected to the wide channel, while 75 μL of plain cell culture medium were loaded into the reservoir connected to the thin channel. The unequal amounts of input liquids enabled to decrease the compound concentration to 25% of the initial concentration. Side by side to the gradually changing concentration profile, MTs were exposed to a constant concentration of 0.75 μM Staurosporine. Control groups were cultured without compound in the chip under identical perfusion conditions and under static conditions in 96-well microtiter plates. The tilting angle was set to 20°, tilting intervals to 2 min 30 s and transition times to 30 s. The chips were then repeatedly tilted. For imaging, the tilting was briefly interrupted, and the plate was transferred onto a microscope. Experiments were performed with at least six MTs per chip and 2–4 chips per condition.

### Biochemical Assays

Intracellular adenosine triphosphate (ATP) was measured using the CellTiter-Glo® 3D Cell Viability Assay (Promega AG, Dübendorf, Switzerland) as a measure of MT viability. The Caspase 3/7-Glo® Assay Systems (Promega AG) was used to assess onset of apoptosis. For both assays, MTs were removed from the chip and transferred into Akura 96-well plates (InSphero AG). MTs were washed with PBS (Life Technologies Europe B.V.), before the assay was performed according to the manufacturer's protocol.

### Imaging

Bright-field images of MTs were recorded using an inverted wide-field microscope (DMI-6000B; Leica Microsystems AG, Heerbrugg, Switzerland), equipped with a CCD camera (DFC-340-FX; Leica) and a stage-top cell culture incubator to maintain cell-culture conditions during the imaging process (Life Imaging Services GmbH, Basel, Switzerland). To focus on the elevated microfluidic chips in the handling frame, a distance ring (Thorlabs GmbH, Dachau, Germany) was used to increase the parfocal length of the objective. All images were analyzed using ImageJ/Fiji software.

## Results

### Theoretical

#### Modulation of the Concentration Profile

The new microfluidic channel design is aimed at reproducing physiologically relevant drug dosing curves *in vitro*. Operation of the chip was intended to be simple and not to rely on additional equipment, such as tubing and pumps. Therefore, the concept of gravity-driven flow by tilting of the chip was applied. Nevertheless, the new design features great flexibility in producing a variety of substance concentration profiles.

Three parameters can be changed to modulate the shape of the produced dosing curve: (i) the dimensions of the channels, (ii) the liquid volumes in the reservoirs at the beginning of an experiment, and (iii) the tilting angle during an experiment. The influence of these three parameters is illustrated in Figure 3A. The hydrodynamic resistance *R*_*S*_ and the initial input volume *V*_*S*_ denote those of the channel containing medium with a compound, while *R*_*M*_ and *V*_*M*_ denote those of the channel with plain medium. The normalized concentration profile curves were calculated using equation (10) after each tilting interval. The initial concentrations were set to 0 in *V*_*M*_ and in the main channel, and 1 in *V*_*S*_.

- Modulation of the channel dimensions (Figure 3D): Equation (4) defines how the flow rates through the channels depend on their hydrodynamic resistance *R*_*h*_. The resistance, in turn, is determined by the channel dimensions, e.g., the channel width (equation 2). Thus, the flow rates through the two channels upstream or left of the Y-junction (Figure 3B) can be individually modulated by adjusting their hydrodynamic resistances *R*_*S*_ and *R*_*M*_ (Figure 3C). The *R*_*S*_-to-*R*_*M*_-ratio influences the mixing ratio of the two liquids originating from the two separate reservoirs. Very large or very small *R*_*S*_*-*to*-R*_*M*_-ratios result in slowly increasing/decreasing concentrations until an equilibrium is reached. The lower the difference of *R*_*S*_ and *R*_*M*_, the shorter and steeper become the concentration curves. For *R*_*S*_/*R*_*M*_ < 1, a curve with decreasing concentrations is produced, while *R*_*S*_/*R*_*M*_ > 1 yields increasing concentrations. Equal flow resistances in both channels result in linear, stable concentrations over time.
- Varying the initial volumes in the reservoirs (Figure 3E): The initially applied liquid volumes *V*_*S*_ and *V*_*M*_ determine the equilibrium concentration at the end of the gradual concentration change, i.e., the dilution of the initial substance concentration. For equal volumes in both reservoirs at the beginning of an experiment, the equilibrium is reached at 50% of the initial substance concentration. For *V*_*S*_/*V*_*M*_ > 1, the concentration profile covers only a small range, before equilibrium is reached at a concentration, which is higher than 50% of the initial substance concentration. For *V*_*S*_/*V*_*M*_ < 1, the opposite holds true. Loading unequal amounts of liquid into the reservoirs also results in different Δ*h* between the lower reservoir at the right side and the two upper reservoirs at the left upon first tilting. The different Δ*h* has an effect on the respective flow through the two channels, which also needs to be taken into account for calculating the flow rates and the concentration profile.
- A variation of the tilting angle of the chip during an experiment can be used to modulate the slope of the dosing curve on demand. The higher the tilting angle, the higher are the flow rates in all channels and, consequently, the steeper becomes the slope of the dosing curve. Figure 3F shows concentration curves at low (~5–10°), medium (~10–30°) and high (~30–50°) tilting angles α, while hydrodynamic resistances and input volumes were kept constant.

The possibility to vary these three parameters leads to a large flexibility in dosing regimens. While the latter two parameters (reservoir filling volume and tilting angle) can be changed during operation, the channel dimensions have to be already defined while designing the microfluidic chip. Practically, the chip has been designed to cover a fairly wide range of dosing curve slopes, which can be fine-tuned by adjusting the input volume and tilting angle to obtain the intended dosing curve.

### Experimental

#### Design Integration Into a Microfluidic Microtissue Culture Chip

**Figure 4:**
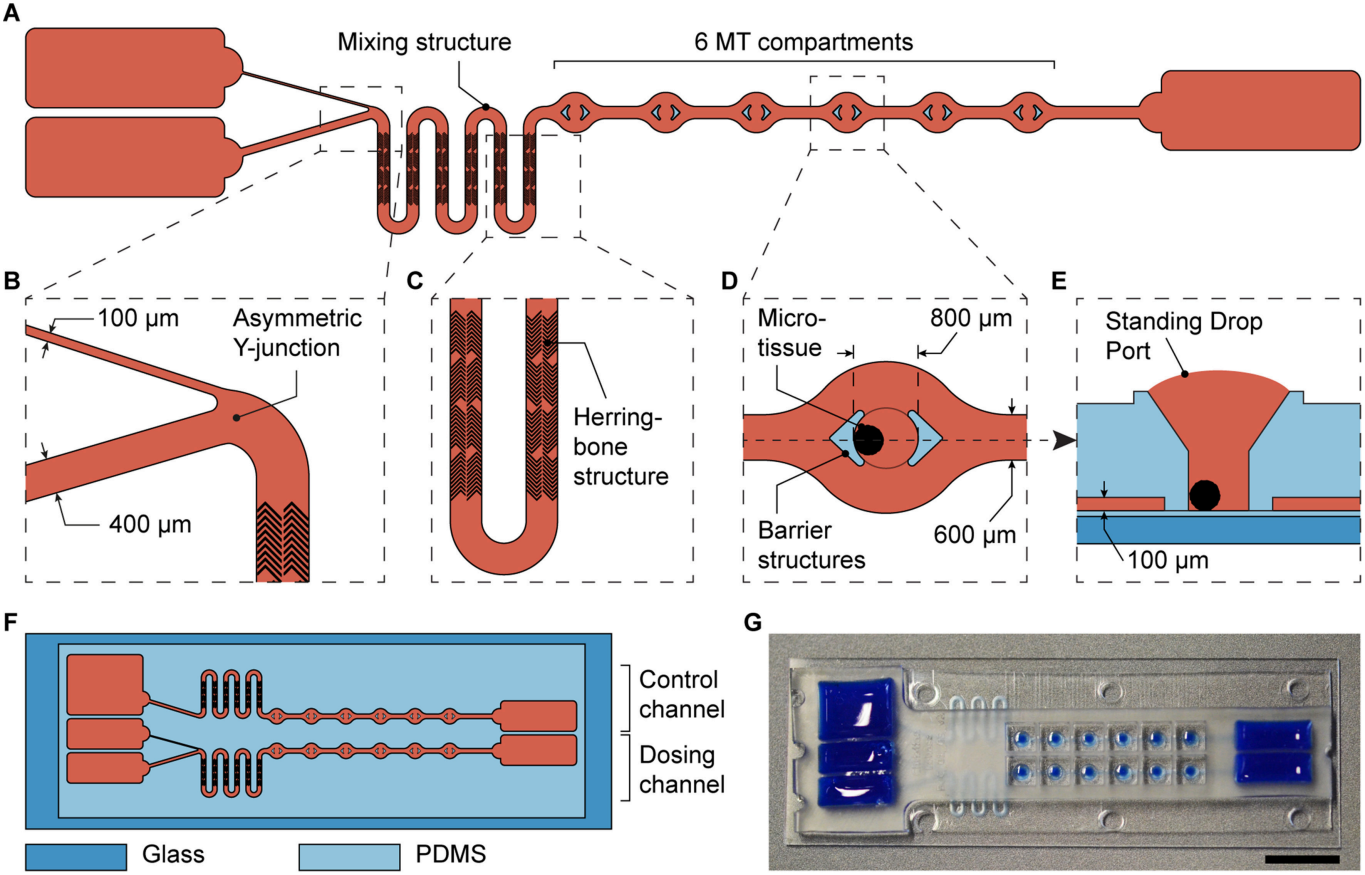
Integration of the asymmetric design into a microfluidic microtissue-culture chip. **(A)** Microfluidic channel layout with the asymmetric Y-junction, followed by a mixing structure and six microtissue (MT) compartments. Details are shown in **(B)** the asymmetric Y-junction, **(C)** the micromixer including herringbone structures in a meander-shaped channel, and **(D)** the top view of a MT compartment with protective barrier structures and **(E)** a side view of a MT compartment. **(F)** Each microfluidic chip has the size of a microscopy slide and includes a dosing channel with a Y-junction, next to a control channel for linear exposure profiles. **(G)** Photograph of the microfluidic chip the channels and reservoirs of which have been filled with a colored liquid (scale bar: 10 mm).

To test the new design, we integrated it into a previously developed microfluidic microtissue culture chip. The original chip features two parallel channels, each with one reservoir at each side and 10 compartments in between, which can be used to culture spherical MTs under constant substance concentrations. The chip is operated by tilting (Lohasz et al., 2018). The basic channel and compartment structure of the chip was maintained. On one side, the left side in Figure 4, a second reservoir and the Y-junction (Figure 4B) were implemented and the number of MT compartments was reduced to six. Further, a mixing structure was added (Figure 4A). As turbulent mixing does not occur at the inherently low Reynolds numbers in microchannels, mixing structures are required to obtain a homogenous substance concentration (Lee et al., 2011). Therefore, we implemented a meander-shaped channel and so-called herring bone structures (Stroock et al., 2002) between the Y-junction and the six MT compartments (Figure 4C). The mixing structure smoothened the sharp spatial gradient originating from the two reservoirs across the channel width to expose all MTs to an almost uniform substance concentration (Supplementary Figure 3 and Supplementary Movie 1). By connecting the chip to syringe pumps and by perfusing it with differently colored solutions, we could show that substance concentrations in all MT compartments change uniformly and in accordance with the concentrations inside the main channel. Furthermore, no differences could be observed between the first and the last MT comportment along the perfusion direction (Supplementary Figure 4 and Supplementary Movie 1).

In addition to the dosing channel with the Y-junction, a control channel was included into each chip. This control channel is similar to the dosing channel, but lacks the Y-junction. Instead, it features a single channel, the design parameters of which were adjusted to obtain similar hydraulic resistances as in the dosing channel, and one reservoir (Figure 4F). The two differently sized channels leading into the Y-junction were 400 and 100 μm wide. The width of the first segment at the left in the control channel was 400 μm wide, and both main channels with MT compartments were 600 μm wide. All channels had a height of 100 μm. Having these two device architectures side by side on the same chip, the effects of a gradually changing concentration profile could be compared to those obtained by applying a linear concentration on the same chip. Twelve MTs were exposed to two different dosing conditions (6 per condition) in each device.

The MT compartments (Figure 4D) are open to the top to facilitate loading of externally produced MTs and their removal after an experiment to be able to individually analyze each MT. Surface tension of the medium generates a liquid-air-interface (*Standing Drop Port*, Figure 4E) defined by the hydrophobic rim structure of the MT compartment. During experimentation, barrier structures hold the MT in place, and protect them from shear stress as a consequence of the liquid flow (Figure 4D). The barrier structures, however, do not hinder mass transport of biomolecules to the MT culturing site, as confirmed by the streamlines in a flow simulation (Supplementary Figure 2C). The details of the MT compartment design and the flow through the compartment have been thoroughly described and characterized previously (Kim et al., 2015b; Lohasz et al., 2018). The MT compartments are 800 μm in diameter and are spaced at a pitch of 4.5 mm. Rectangular reservoirs at the ends of the channels contain up to 150 μL per channel. The chip was fabricated using PDMS, and had a total size of 25 × 75 mm.

#### Concentration Profile Characterization

We designed a chip that could be used to deliver approximately typical, decreasing blood-plasma concentrations of substances that are administered to patients by injection (Kwon, 2001). Injection of compounds generally results in initially high-concentration peaks, followed by a decrease over time (Figure 1). For the two channels at the left or upstream of the Y-junction in the dosing device, we used 400 μm width and 100 μm width.

**Figure 5:**
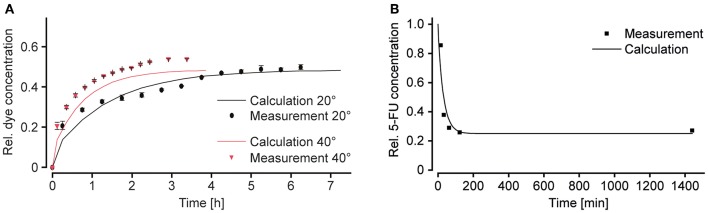
Modulation of the substance concentration profile. **(A)** Amaranth red dye was used to simulate and track substance concentrations in the chip over time. Changing the tilting angle α modulates the slope of the concentration curve (*V*_*S*_/*V*_*M*_ = 1; *R*_*S*_/*R*_*M*_ = 4; *n* = 3 chips). **(B)** Decreasing concentration of 5-fluorouracil (5-FU) in the main channel over time. Peak area and concentration measurements were done by means of ultra-performance liquid chromatography, coupled to mass spectrometry UPLC-MS (*V*_*S*_/*V*_*M*_ = 0.66; *R*_*S*_/*R*_*M*_ > 0.25; α = 20°; *n* = 1 chip).

We demonstrated the flexible modulation of the concentration profile on demand upon changing the tilting angle α (Figure 5A) through concentration measurements of amaranth red dye in the shared reservoir at the right side. Dye solution was loaded into the reservoir connected to the narrow channel, while plain medium was loaded into the other one. Repeated tilting resulted in a gradual increase of the dye concentration within the main channel and the shared reservoir. Figure 5A shows the measured curves for tilting angles of 20 and 40° until a concentration equilibrium was reached (after 6.5 h at 20° and after 3.5 h at 40°). The curve was also compared to calculated curves for the respective chip dimensions and a *V*_*S*_-to-*V*_*M*_-ratio of 1. The measured concentrations matched the calculated values, however, with a small offset. Furthermore, the curve, obtained with a tilting angle of 20°, showed minor fluctuations between 2 and 4 h. Both features can be explained by medium evaporation due to opening of the chip system at 15-min intervals for sampling. The results show that the concentration increase to a steady state concentration is faster with a steeper slope at higher tilting angles. A modulation of the shape of the dosing curve by changing the tilting angle α was indeed possible.

For obtaining decreasing concentration profiles, the wide channel was used for the substance-containing medium, and the narrow channel for plain medium. By interchanging the two liquids in the reservoirs, as compared to the first characterization experiment, a *R*_*S*_-to-*R*_*M*_-ratio > 1 was achieved. Decreasing concentration profiles could be used to reproduce physiologically relevant conditions upon injection of a compound. For this second characterization experiment, 5-fluorouracil (5-FU) was used. 5-FU is a commonly used anti-cancer drug, which is administered by infusion or injection (Casale et al., 2004). Tilting was started with an angle of 20°, and samples were taken from the shared reservoir at the right side after 15, 33, 63, and 123 min, as well as after 24 h. 5-FU concentrations were measured by mass spectrometry (Figure 5B). The measured points were in good agreement with the calculated concentration profile and a steeply decreasing dosing curve was produced. A steady state at 25% of the initially applied concentration could be reached by applying a *V*_*S*_-to-*V*_*M*_-ratio of 0.66. The steady state was reached after ~2 h of continuous tilting of the chip.

The results of the two characterization experiments showed that concentration profiles can indeed be modulated by changing parameters, such as tilting angles (Figure 5A), resistances of the channels, or input volumes in the reservoirs (Figure 5B). One major difference between the concentration profiles produced with the device and the ones found *in vivo* is that the final equilibrium substance concentration obtained with the microfluidic chip is defined by the two initial concentrations and volumes in the reservoirs at the left and is always larger than 0, whereas the substance is eliminated completely in the human body in case of a single dosage. A possible solution to also reach a concentration of 0 with the microfluidic chip could include medium exchanges after the intended exposure time. Moreover, most substances are, to a certain amount, metabolized by the respective target tissue, which—depending on the initial substance concentration—might also contribute to decreasing the final concentration to physiological levels.

According to the MT compartment design, all MTs sit under a vertical liquid column of ~1 μL, which is not fully subjected to active perfusion from the channels. Alterations of the exposure profile by diffusion effects originating from these liquid columns, however, were considered negligible. This assumption is based on the small volume of the liquid columns in comparison to the continuous active perfusion with flow rates of 10–25 μL min^−1^, depending on the tilting angle.

All in all, the proposed chip features large flexibility to produce concentration profiles that resemble those of *in vivo* scenarios upon injection of a substance. Concentration curves in the chip system could reliably be predicted and reproduced.

#### Dosing Experiments

**Figure 6:**
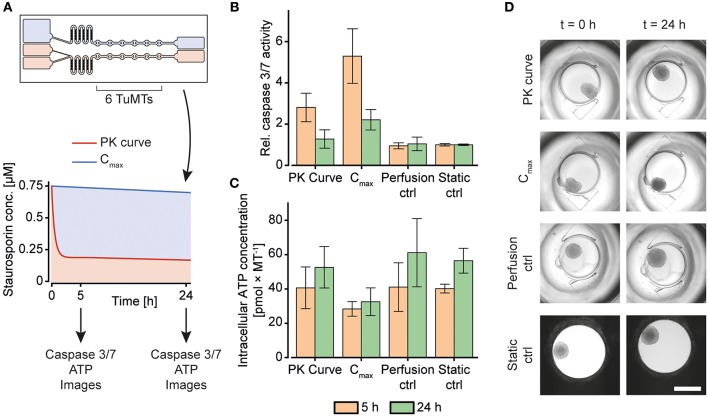
Staurosporine treatment of human colorectal tumor (HCT 116) microtissues (TuMTs) in the microfluidic chip. **(A)** Experimental layout: A chip was used to expose six TuMTs per channel to a PK-like dosing profile or a constant dose. The initial maximum concentration (C_max_) of the PK-like profile was chosen as the constant concentration for the second channel. The perfusion control and the static control MTs were cultured without any compound treatment in the chip and in a 96-well microtiter plate, respectively. Readouts were taken 5 and 24 h after treatment start. **(B)** Relative caspase 3/7 activity, normalized to those of the static control of the respective sampling time points (data represented as mean ± SD; *n* = 6–12 TuMTs). **(C)** Intracellular ATP concentration as a measure of viability (data represented as mean ± SD; *n* = 6–12 TuMTs). **(D)** Representative images of TuMTs on the chip and in the microtiter plate at 0 and 24 h (scale bar: 500 μm).

After the new system had been characterized, it was tested by exposing a tumor MT (TuMT) model to a gradually decreasing, PK-like dosing profile, and, for comparison, to a constant dose (Figure 6A). We used the human colorectal cancer-cell line HCT 116 as a suitable tumor model, based on (i) the possibility to reliably generate uniform TuMTs, and (ii) its sensitivity to Staurosporine. Staurosporine is an anti-cancer tool compound, commonly used for *in vitro* studies. It acts as a potent, promiscuous kinase inhibitor and induces apoptosis in proliferating cancer cells (Chae et al., 2000; Karaman et al., 2008). The dosing curve that was previously characterized with 5-fluorouracil (5-FU) was applied to the TuMTs over a duration of 24 h. The initial maximum concentration (C_max_) of the PK-curve was chosen as constant concentration for comparison. A concentration of 0.75 μM was determined as C_max_ by a static titration prior to the dosing experiment (Supplementary Figure 5).

After 5 h and 24 h of incubation, caspase 3 and 7 activity was measured to assess the induction of apoptosis as a reaction to the different Staurosporine treatment strategies (Figure 6B). After 5 h of treatment, TuMTs under both treatment conditions showed increased caspase 3/7 activity, as compared to the untreated controls. Moreover, TuMTs treated constantly with the C_max_ concentration showed higher levels of apoptosis. Measurements of apoptosis after 24 h indicated slightly increased caspase activity for the C_max_ condition, while caspase levels were similar to those of the control groups for the PK-like dosing regimen. Figure 6C shows intracellular ATP concentrations of the TuMTs as a measure of viability after 5 h and 24 h. The increasing ATP concentrations for all conditions over time indicate proliferation of the cancer cells in the TuMTs. While ATP concentrations in the PK-treated and the control MTs increased to a similar extent, they remained almost constant for the C_max_-treated MTs. Bright-field images of the conditions at the start of the experiment and after 24 h confirm the ATP findings, as they show slight MT growth for all conditions, except for the C_max_-treated ones (Figure 6D). Furthermore, a constant high dose of Staurosporine led to a morphological change of the TuMTs, which appeared darker and denser after 24 h of treatment. Noteworthy, the switch from static to perfusion culture did not affect cell viability and health, as can be seen by comparing the perfusion control to the static control (Figures 6B,C). This observation indicates that flow-induced shear stress on the TuMTs does not compromise their characteristics and behavior, and it evidences that the developed device is suitable for assessing effects of pharmacokinetic substance profiles on TuMTs.

The acquired data indicated, that the different treatment conditions with the same starting concentration may, indeed, yield different results. While the anti-cancer effect of Staurosporine lead to an early onset of apoptosis for the PK-profile, the mid-term and long-term effect was more prominent for the constant high dose. The experiments highlight that a certain threshold concentration of an active compound has to be maintained over the treatment duration in order to obtain the desired effect. Metabolization effects of administered compounds as they occur *in vivo*, however, may decrease the concentration even more rapidly so that it may be difficult to maintain the concentration within the therapeutic window. In any case, it is advantageous for an *in vitro* system that is intended to reproduce the *in vivo* situation as much as possible to have options to adapt the dosing profile to accommodate various effects and maintain critical concentrations.

## Discussion

We presented in this article a technical solution for the exposure of cellular models to temporal gradients of a substance of interest. The concentration profile was achieved by an asymmetrical Y-junction in a microfluidic microtissue culture chip that relied on perfusion by gravity-driven flow. We did not use external pumps and tubing, and we applied SBS Standard ANSI/SLAS 4-2004^1^ for the MT compartments and Standard ANSI/SLAS 1-2004^2^ for the chip handling frames, so that our platform meets all criteria for parallelization and automation of experiments.

Other systems featuring dynamic exposure profiles of cellular model systems have been presented in the last couple of years. Song et al. (2018) presented a system to expose HEK cells to short pulses and gradually ramped profiles of epidermal growth factor (EGF). Their pump-driven microfluidic chip enabled to optically monitor cellular responses at single-cell resolution. Differential pathway activation was detected in an exposure-profile-dependent manner. The application of such pump-driven systems, however, requires technically experienced operators, which limits their broad use, experimental throughput, and the transfer of such systems to non-expert labs.

Another approach for pulsatile substance exposure of cellular models, was realized by relying on passive, gravity-driven perfusion (Lee et al., 2016). The proposed microfluidic chip was connected to two inlet reservoirs at different heights, one filled with medium, and the other one filled with substance-containing medium. In their work, the authors cultured cancer cells in a microfluidic chip and exposed them to pulses of tumor necrosis factor (TNF). Pulses were initiated by manually elevating the reservoir containing the substance for the duration of the pulse. Interestingly, their results evidenced higher anticancer effects for using shorter pulses of TNF, which underline the importance of investigating dosage-dependent toxicity and efficacy. The system, however, was only used to generate pulses of TNF, and gradual concentration changes over time were not realized. The system presented here, on the other hand, can be used to produce gradual changes of substance concentrations without the need for manual actuation, while still fully relying on gravity-driven operation. It can be used with a minimum set of specialized equipment. Furthermore, in contrast to other systems, which were designed for culturing 2D monolayers of cells, our chip was devised to culture multiple 3D MTs.

Combining a controlled microfluidic environment with 3D cell culture techniques to enable the realization of PK profiles and modification of the respective parameters, we see great potential for our design for investigating concentration profile-dependent efficacy and/or toxicity of substances.

### Future Perspective

As shown in the presented experiments, different concentration profiles could be produced in the microfluidic chip, and tumor MTs could be exposed to dynamically changing concentrations profiles. So far, the chip was used for experimentation with only one type of MTs. The chip, however, is not limited to a single organ surrogate, but also allows for combining several different MTs or organ types. The behavior of different organs upon substance exposure can be monitored simultaneously, which allows for a combined readout of efficacy on the primary target organ and toxicity on a secondary organ. For mimicking cancer treatment, tumor MTs and liver MTs could be combined to assess the side effects of tumor treatment on the liver. The developed device could help to experimentally modify the dosing curve in order to find a dosing strategy with an optimum trade-off between efficacy on the tumor and liver toxicity. Moreover, communication between the two tissues types through secreted molecules, which might further influence their drug and treatment response, could be included in *in vitro* testing.

**Figure 7:**
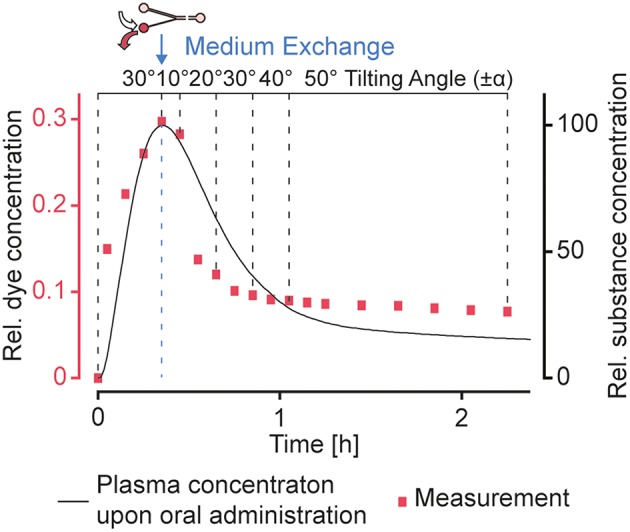
Realization of more complex dosing profiles. A typical concentration profile (Kwon, 2001) upon oral administration of a substance could be approximated by using a sequence of different tilting angles. A medium exchange to replace the substance-loaded medium in the reservoir connected to the narrow channel with plain medium was performed after ~20 min. Amaranth red dye was used as drug-surrogate.

In addition to the presented concentration profiles, more complex dosing profiles could be approximated by applying a sequence of different tilting angles and precisely timed medium changes in the reservoirs. We could produce a concentration profile that features the characteristics of a typical plasma concentration over time upon oral administration of a substance (Kwon, 2001), as we have demonstrated by using amaranth red dye as drug-surrogate (Figure 7). A sequence of precisely timed medium exchanges, or the implementation of capillary stop valves that burst when sufficient pressure is built up at high tilting angles (Olanrewaju et al., 2016, 2018) can be used over longer culturing periods to reproduce long-term substance fluctuations, e.g., repeated drug dosing or circadian hormone profiles. Alternating filling of the reservoir connected to the narrow channel with hormone-containing and plain medium could be used to gradually increase and decrease hormone concentrations in the MT compartments. Since circadian profiles modulate organ functions, they may play an important role in future substance testing and for translating of *in vitro* organ responses to *in vivo* conditions (Cyr et al., 2017).

## Conclusion

In this article, we presented a novel microfluidic design for the generation of gradually changing substance concentration profiles for an easy-to-use microfluidic tilting system. The integration of the new design into cell culture chips enabled to run dynamically changing concentration profiles and to reproduce, e.g., the dosing curve upon injection of a substance without the need for a series tedious pipetting steps or expensive pumping equipment. By changing design and operation parameters, the dosing curves could be adjusted to experimenters needs, and the dosing curves could be modulated on demand as demonstrated by recreating an oral uptake profile. Experiments with colorectal tumor MTs and the cytotoxic substance Staurosporine showed the different short- and long-term responses of the MTs to dynamic and traditional, linear dosing regimes.

## Author Contributions

CL, OF, and KR conceived the approach and designed the experiments. CL and FB performed the experiments. CL analyzed the data. CL, OF, KR, and AH wrote the paper.

### Conflict of Interest Statement

OF is part of the management team at InSphero AG commercializing microfluidic culturing devices. The remaining authors declare that the research was conducted in the absence of any commercial or financial relationships that could be construed as a potential conflict of interest.

## Footnotes

^1^ANSI/SLAS 1-2004: Microplates–Footprint Dimensions.

^2^ANSI/SLAS 4-2004: Microplates–Well Positions.
