# Supplementary material for: Tubing-Free Microfluidic Microtissue Culture System Featuring Gradual, in vivo-Like Substance Exposure Profiles
Source: Front Bioeng Biotechnol. 2019 Apr 2;7:72. doi: 10.3389/fbioe.2019.00072 (PMC6454105; doi:10.3389/fbioe.2019.00072)
Supplement: Supplementary file 1 [file Data_Sheet_1.pdf]

Supplementary Material

Tubing-free microfluidic microtissue culture system featuring gradual, in vivo-like substance exposure profiles

Christian Lohasz[1*], Olivier Frey[2], Flavio Bonanini[1], Kasper Renggli[1], Andreas Hierlemann[1]

[1]Bioengineering Laboratory, Department of Biosystems Science and Engineering, ETH Zurich, Switzerland

[2]InSphero AG, Schlieren, Switzerland

***Correspondence:** Christian Lohasz, christian.lohasz@bsse.ethz.ch

1 Supplementary Figures

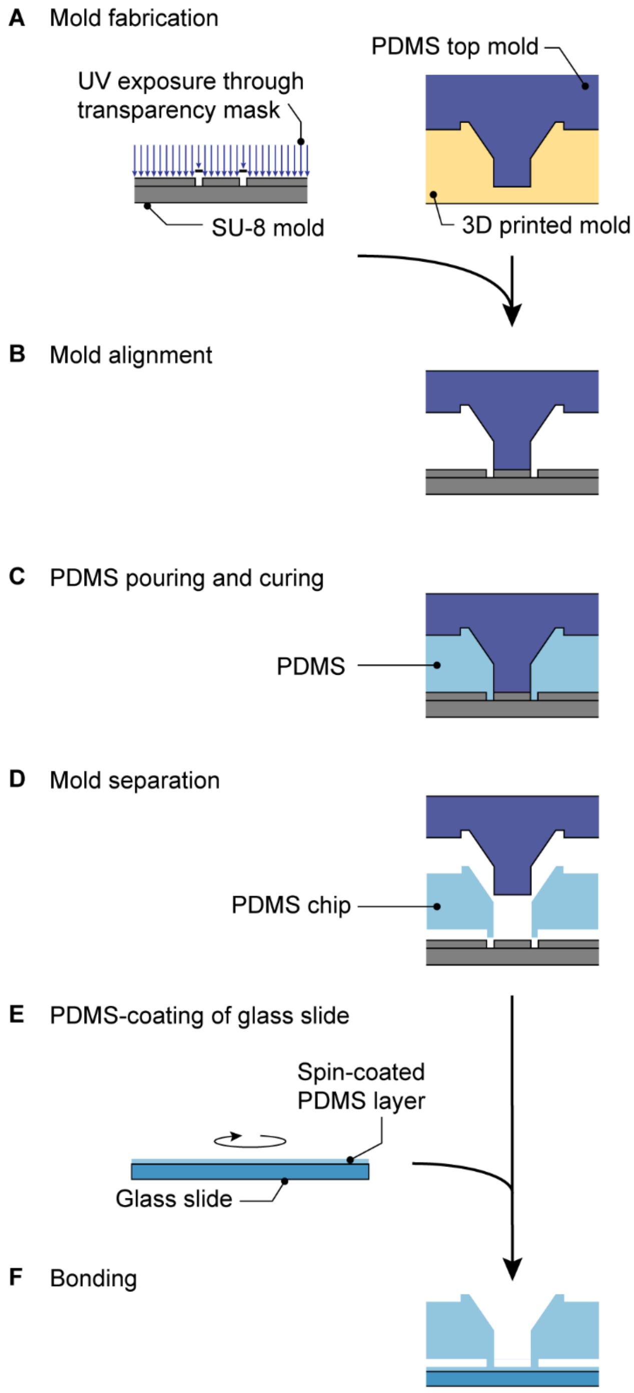

Supplementary Figure 1: Fabrication of the microfluidic chip exemplified for a MT compartment. **(A)** An SU-8 bottom mold was fabricated using standard photolithography methods, and a PDMS top mold was casted using a 3D-printed mold. **(B)** The bottom and top molds were aligned, and **(C)** liquid PDMS was poured between them. **(D)** After PDMS curing, the re-usable molds were separated. **(D)** Microscopy glass slides were spin-coated with a uniform PDMS layer, and **(F)** the chips were O_2-plasma-bonded onto those slides.

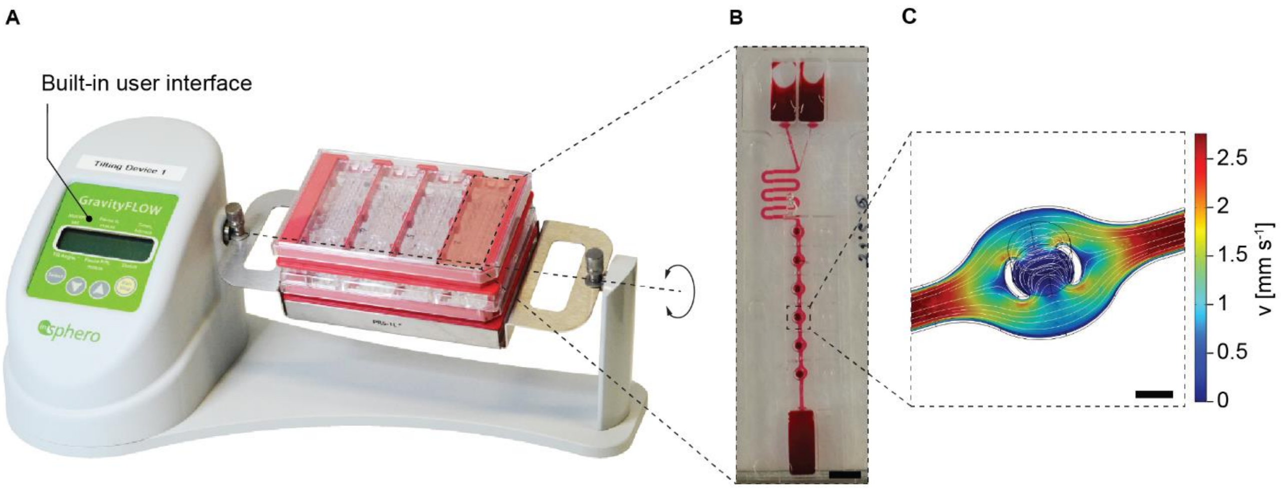

Supplementary Figure 2: Operation of the microfluidic microtissue culture chip by tilting. **(A)** Four chips are assembled in a handling frame, and several handling frames can be stacked onto a programmable tilting stage with a built-in user interface that allows for setting tilting angles, duration of the resting times at tilted positions, and the transition times between positive and negative tilting angles. **(B)** Image of a chip on the tilting stage. Channels of the chip were filled with dye for illustration (scale bar = 5 mm). **(C)** Assessment of the liquid velocity around the microtissue compartment to estimate shear stress and compound delivery into the MT compartment at a flow rate of 10 µL min^{-1}. Stream lines indicate mass transport of biomolecules into the MT compartment (scale bar = 500 µm).

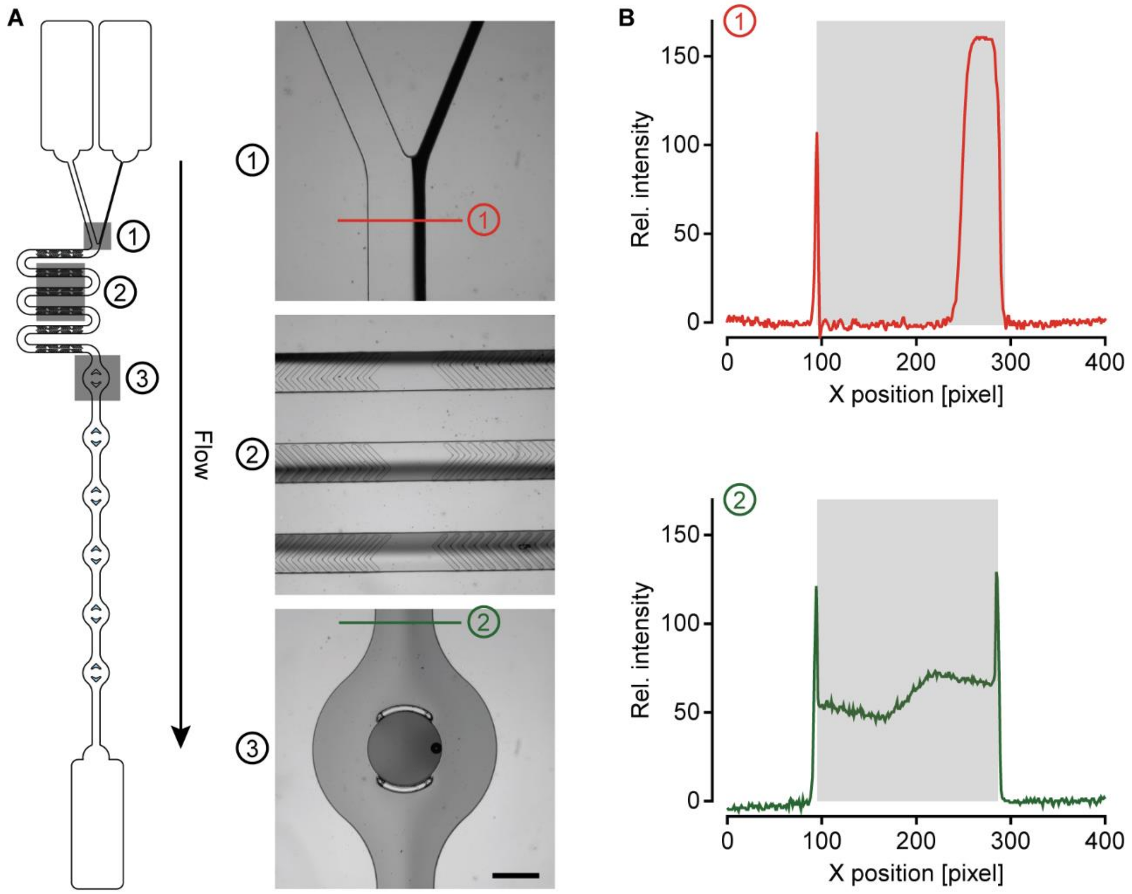

Supplementary Figure 3: Efficiency of the mixing element in the microfluidic chip. **(A)** Microfluidic channel layout and microscopy bright-field images of the indicated positions. An amaranth red-dye solution was flushed through the narrow channel, while water was flushed through the other one. Along the mixing structure, the two liquid phases mix, before they reach the microtissue compartments (scale bar: 500 µm). **(B)** Relative greyscale intensity along the indicated lines in the microscopy images. After the mixing structure, amaranth red dye was homogeneously distributed across the entire channel width.

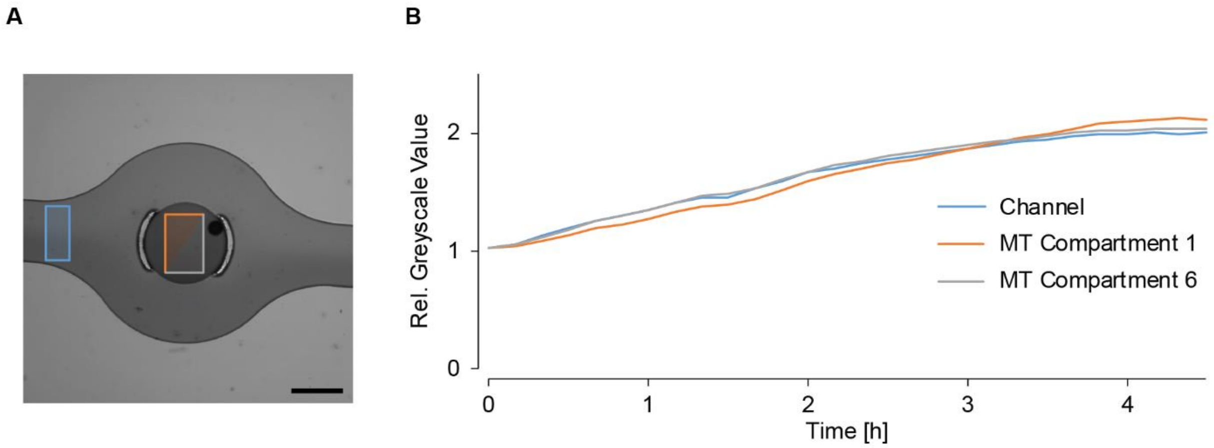

Supplementary Figure 4: Concentration change within the channel and the MT compartments over time. **(A)** Microscopic image of a MT compartment under perfusion with amaranth red dye and water from two separate inlets (see also Supplementary Movie 1). Rectangles indicate regions of interest for the channel and the MT compartments (scale bar: 500 µm). **(B)** Normalized mean grey-scale values in the channel, the first MT compartment, and the last MT compartment along the perfusion direction over time as an indicator for changing dye concentrations. The amaranth red concentration is steadily increasing over time, while the flow rate was kept constant at 15 µL min-1. Images were taken at 10 min intervals.

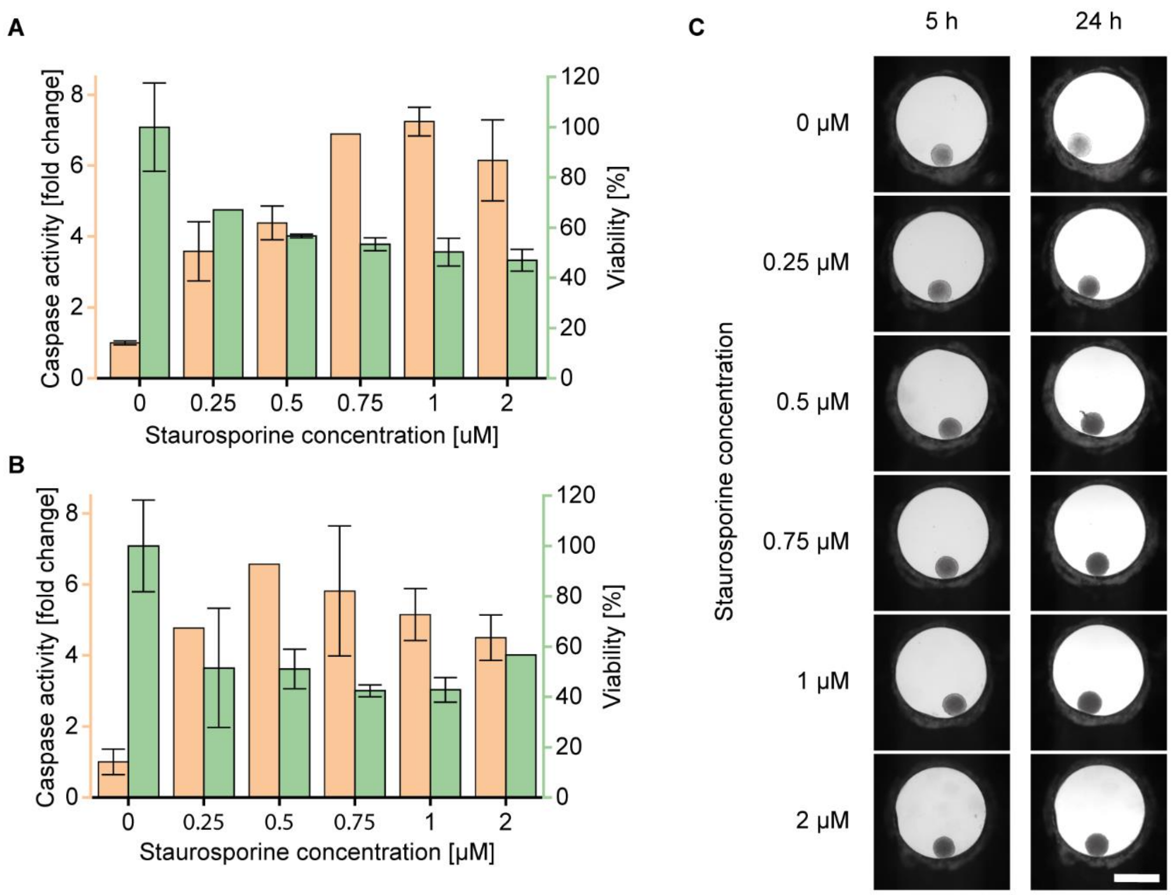

Supplementary Figure 5: Titration of Staurosporine with TuMTs under static conditions in a 96-well microtiter plate. Caspase 3/7 activity and ATP-dependent viability were measured **(A)** 5 h and **(B)** 24 h after treatment start. A dose-dependent increase of caspase induction and a decrease of viability could be observed up to a dose of 0.75 µM. Higher doses resulted in a response similar to 0.75 µM (data represented as mean ± SD). **(C)** Images of TuMTs appear darker and denser upon Staurosporine treatment, slight disintegration is observed after 24 h (scale bar: 500 µm).

2 Supplementary Movie

Supplementary Movie 1: Different regions of the microfluidic chip under perfusion with amaranth red dye and water from two separate inlets. The amaranth red concentration is steadily increasing over time, while the flow rate was kept constant at 15 µL min^{-1}. Images were taken at 10 min intervals.
